# Guidelines for safe handling of hazardous drugs: A systematic review

**DOI:** 10.1371/journal.pone.0197172

**Published:** 2018-05-11

**Authors:** Mari A. Bernabeu-Martínez, Mateo Ramos Merino, Juan M. Santos Gago, Luis M. Álvarez Sabucedo, Carmina Wanden-Berghe, Javier Sanz-Valero

**Affiliations:** 1 Department of Public Health and History of Science, School of Medicine, Miguel Hernandez University, Elche, Spain; 2 Department of Telematics Engineering, Telecommunication Engineering School of the University of Vigo, Vigo, Spain; 3 Health and Biomedical Research Institute of Alicante, University General Hospital of Alicante, Alicante, Spain; University of South Alabama Mitchell Cancer Institute, UNITED STATES

## Abstract

**Objective:**

To review the scientific literature related to the safe handling of hazardous drugs (HDs).

**Method:**

Critical analysis of works retrieved from MEDLINE, the Cochrane Library, Scopus, CINHAL, Web of Science and LILACS using the terms "Hazardous Substances", "Antineoplastic Agents" and "Cytostatic Agents", applying "Humans" and "Guidelines" as filters. Date of search: January 2017.

**Results:**

In total, 1100 references were retrieved, and from those, 61 documents were selected based on the inclusion and exclusion criteria: 24 (39.3%) documents related to recommendations about HDs; 27 (44.3%) about antineoplastic agents, and 10 (33.3%) about other types of substances (monoclonal antibodies, gene medicine and other chemical and biological agents). In 14 (23.3%) guides, all the stages in the manipulation process involving a risk due to exposure were considered. Only one guide addressed all stages of the handling process of HDs (including stages with and without the risk of exposure). The most described stages were drug preparation (41 guides, 67.2%), staff training and/or patient education (38 guides, 62.3%), and administration (37 guides, 60.7%). No standardized informatics system was found that ensured quality management, traceability and minimization of the risks associated with these drugs.

**Conclusions:**

Most of the analysed guidelines limit their recommendations to the manipulation of antineoplastics. The most frequently described activities were preparation, training, and administration. It would be convenient to apply ICTs (Information and Communications Technologies) to manage processes involving HDs in a more complete and simpler fashion.

## Introduction

The toxic properties of cytostatic drugs have been well known since the 1940s when they began to be used in the oncological field [1]. However, it took nearly four decades before Falck et al. [2] published the first paper describing an increase in mutagenicity in nurses working with cytostatic drugs, demonstrating for the first time the potential occupational risk involved in the manipulation of these medicines. The publication of a series of subsequent studies [3–6], whose results pointed to the possible relationship between occupational exposure to cytostatics and the increase of various health effects, was key for different government organizations and scientific societies to establish the first guidelines for the safe handling of this type of medication. In 1981, the Society of Hospital Pharmacists of Australia (SHPA) published the first guide for the safe management of cytostatic medicines [7], and four years later, their North American colleagues followed suit [8, 9].

The concept of a "hazardous drug" (HD), which until then was exclusively associated with cytostatic drugs, was introduced in 1990 by the American Society of Hospital Pharmacists (ASHP) [10] and adopted in 2004 by the National Institute for Occupational Safety and Health (NIOSH). This led to the current and internationally accepted definition: any medicinal product that presents in humans one or more of the following hazard criteria: carcinogenicity, teratogenicity or other developmental toxicity, reproductive toxicity, low dose organ toxicity, genotoxicity or drugs with a similar structure or toxicity profile to other dangerous drugs [11].

Later, in 2014, the NIOSH classified HDs into three groups [12]: antineoplastic drugs; non-antineoplastic drugs that meet at least one criterion of danger; and drugs that present a risk to the reproductive process and which may affect men and women who are attempting to conceive actively and to pregnant or lactating women, but do not pose any risk to the rest of the population.

Hazardous drugs, specifically the subgroup of antineoplastic drugs, have been described as the greatest chemical hazard present in the health field and one of the most dangerous chemical agents ever developed [13].

Organizations focused on occupational safety, such as the Joint Commission [14], the Occupational and Safety and Health Administration (OSHA) [15], the Pan American Health Organization (PAHO) [16] and the European Agency for Safety and Health at Work (EU-OSHA) [17], are paying increasing attention to recommendations and strategies for improving safety regarding HDs.

Importantly, given the complexity and interdisciplinary nature of HD manipulation, these processes are particularly error prone. This fact, in addition to the inherent hazards already described, leads us to consider HD as a high-risk therapy that can pose serious risks for both the patient and the involved professionals [18,19].

Therefore, it is essential to standardize these processes because when a protocol correctly implements clinical guidelines, the variability is reduced. This leads to improved quality and minimized risks associated with this type of medication [20].

However, despite efforts made over the past four decades at the international level to establish guidelines to ensure the safe use of HDs, there are currently no globally harmonized standards for the prevention of HD exposure [13], and the ever-worrisome problem is far from being solved [13,21].

For all the reasons abovementioned, it seems mandatory to achieve an updated revision of the main recommendations and/or standards related to the manipulation of HDs. To achieve a standardized model to handling HDs, the main stages involved in proper HD manipulation must be identified, as should preventive measures that can be applied to avoid occupational exposure to HDs. Therefore, the objective of this work was to review the scientific literature on the safe handling of HDs.

## Materials and methods

### Design

A descriptive cross-sectional study and critical analysis of the works recovered through systematic techniques was conducted.

### Sources of data collection

The data were retrieved from direct query and access, on the Internet, from the following bibliographic databases in the field of health sciences: MEDLINE (via PubMed), The Cochrane Library, Scopus, Cumulative Index to Nursing and Allied Health Literature (CINHAL), Web of Science (ISI-Institute for Scientific Information) and LILACS.

### Information processing

To define the search terms, the Medical Subject Headings (MeSH), a *thesaurus* developed by the U.S. National Library of Medicine, was used. The MeSH descriptors "Antineoplastic Agents", "Hazardous Substances" and "Cytostatic Agents" were considered suitable. Likewise, these terms were used to query the database using the title and abstract field (Title/Abstract).

The main search strategy was created for its usage in the MEDLINE database, via PubMed, using the filters "Humans" and "Guidelines", S1 File.

The search was restricted to results from September 2004 (date of the first NIOSH alert, which establishes the current internationally accepted definition of HD) until January 2017 (moment of the last update). This strategy was adapted to the particular features of other databases considered.

Additionally, a search using a complementary strategy was conducted to reduce the possibility of publication bias by searching the reference lists of relevant guidelines. Furthermore, experts in the domain were contacted to avoid issues regarding possible grey literature (materials and research produced by organizations outside of the traditional commercial or academic publishing and distribution channels).

### Inclusion and exclusion criteria

**Fig 1:**
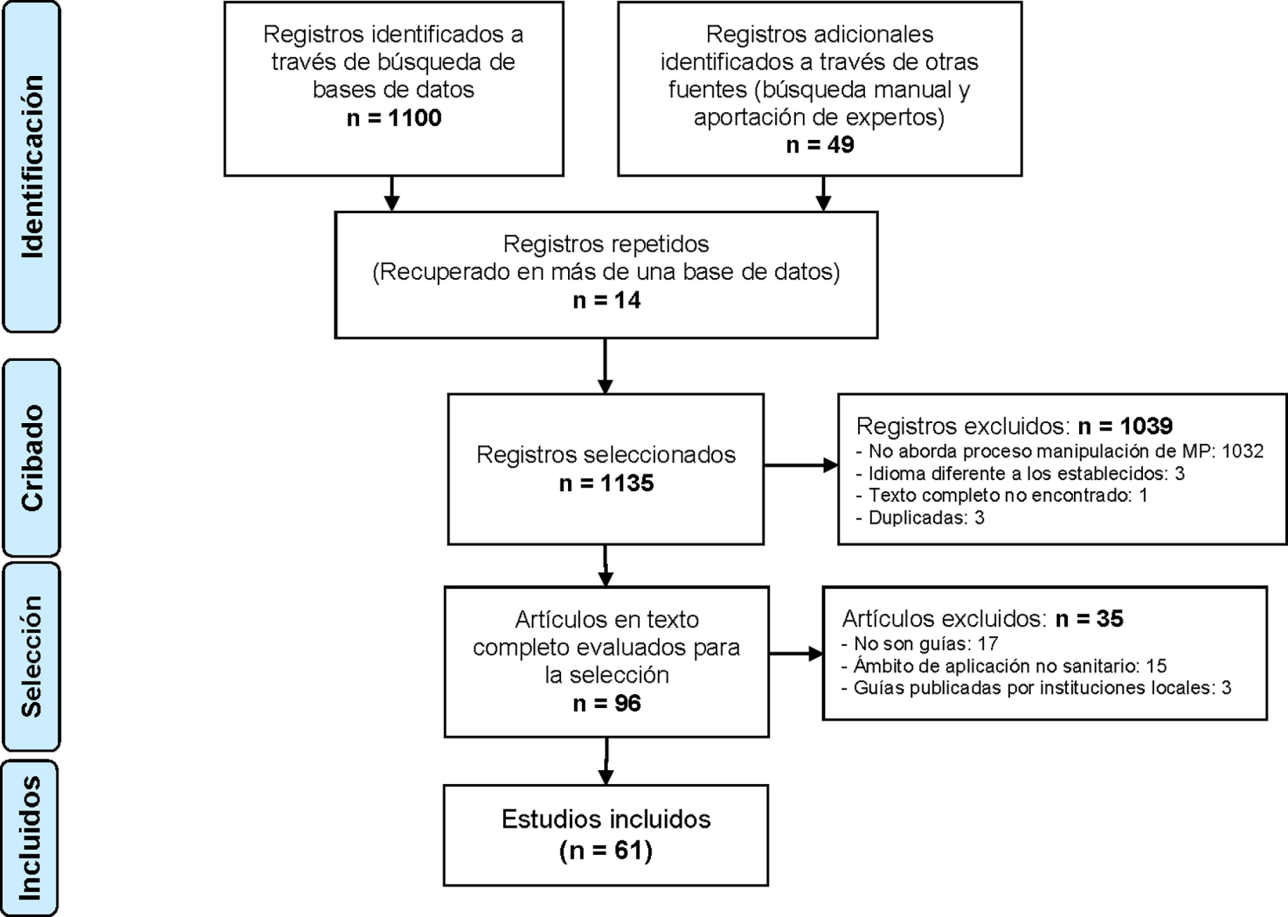
Identification and selection of studies.

The records were subsequently screened according to the a priori inclusion and exclusion criteria shown in Fig 1.

#### Inclusion criteria (I)

Articles that dealt with the HD handling process (I1) and were published in English or Spanish (I2). Additionally, the full text of the document should be accessible (I3). Only one version of each document was included (R). The same criterion was applied to those documents that were duplicated (I4).

#### Exclusion criteria (E)

Documents whose scope of application was not health (E1) and all those published by local institutions (E2). Moreover, works were excluded that could not be considered guidelines (E3) according to the definition by MeSH (i.e., a set of statements, directions, or principles presenting current or future rules or policies. Guidelines may be developed by government agencies at any level, institutions, organizations such as professional societies or governing boards, or by the convening of expert panels). It must be noted that many guidelines are presented to the reader as recommendations although they fit in the former definition by MeSH.

### Final selection of articles

The selection of relevant articles was performed independently by two authors: MBM and JSV. To validate the inclusion of the studies, the assessment of concordance between these authors (Kappa index) should be higher than 80% [22]. Whenever this condition was not met, the possible discordances were solved by consulting the author CWB and subsequent consensus among all the authors.

### Data extraction

The continuous control of the validity of the data was ensured using double tables that allowed detection and corrections of errors by means of new queries to the original data. The Burton-Kebler half-period (the median age) and the Price Index (percentage of the articles published in the last 5 years) were calculated to determine the actuality of the articles.

The chosen documents were classified to systematize and facilitate the understanding of the results and were collected in a table showing the most relevant information from each work. In particular, the following variables were included [Table 1]: first author of the bibliographical reference and year of publication, country, institution or organization that developed the guide, type of institution (governmental, non-governmental, or professional), type of hazardous substance being addressed (HD (based on the NIOSH 2004 alert), antineoplastic (refers to anticancer drugs) and other substances), scope (institution or place where HD is applied), and stages of the process being controlled:

1. Stages with risk of exposure: reception and storage, drug preparation, transportation and distribution, administration, extravasation, patient care (excreta handling, body fluids, and linen), waste management, procedures in case of spill or accidental exposure, and cleaning procedures.
2. Stages without risk of exposure: selection of medicines and commercial presentations (choice of medicines at the time of purchase, taking into account specific aspects that may affect the safety and health of professionals, patients and the environment, such as robust packaging to prevent breakage, design that minimizes handling, etc.), staff training and/or patient education, documentation, medical surveillance and environmental and/or biological monitoring of hazardous substances, understood as the measurement of chemical substances and their metabolites in exposed workers [23].
3. Complementary stages: prescription, validation, patient monitoring, manufacturing by the industry and sterilization.

**Table 1:** Description of the articles selected for review.

Article	Country	Institution*	Institution type	Language	Type of hazardous substance	Scope	Control stages
Neuss *et al*., 2017 [24]	USA	ASCO/ONS	Professional	English	Antineoplastic drugs	Healthcare centres	Stage a: receiving and storage, drug preparation, transport, administration, extravasation (as a complication of administration), patient care. Stage b: training, documentation. Stage c: prescription.
OSHA, 2016 [15]	USA	OSHA	Governmental	English	Hazardous drugs	Healthcare centres	Stage a: receiving and storage, transport, drug preparation, administration, waste management, cleaning procedures, accidental exposure and spill control, patient care. stage b: training, medical surveillance, documentation, biological and environmental monitoring.
Connor *et al*., 2016 [25]	USA	NIOSH	Governmental	English	Hazardous drugs	Healthcare centres	Stage a: receiving and storage, drug preparation, administration, waste management, cleaning procedures, patient care, accidental exposure and spill control.
Poveda *et al*., 2016 [26]	Spain	SEFH	Professional	Spanish	Hazardous drugs	Healthcare centres	Stage a: receiving and storage, drug preparation, transport, administration, waste management, cleaning procedures, patient care, accidental exposure and spill control; stage b: training, medical surveillance, documentation, drug selection.
Delgado *et al*., 2016 [27]	Spain	INSHT	Governmental	Spanish	Hazardous drugs	Healthcare centres	Stage a: drug preparation, administration.
Lepe *et al*., 2016 [28]	Spain	Conselleria de Sanitat Universal i Salut Pública, GV	Governmental	Spanish	Hazardous drugs	Healthcare centres	Stage a: drug preparation, transport, waste management, cleaning procedures, accidental exposure and spill control; stage b: training.
Lepe *et al*., 2016 [29]	Spain	Conselleria de Sanitat Universal i Salut Pública, Generalitat Valenciana	Governmental	Spanish	Hazardous drugs	Healthcare centres	Stage a: drug preparation, transport, administration, waste management, cleaning procedures, accidental exposure and spill control; stage b: training.
García Salom *et al*., 2016 [30]	Spain	Conselleria de Sanitat Universal i Salut Pública, Generalitat Valenciana	Governmental	Spanish	Hazardous drugs	Healthcare centres	Stage a: receiving and storage and drug preparation (facilities).
USP Convention, 2016 [31]	USA	USP	Governmental	English	Hazardous drugs	Healthcare centres	Stage a: receiving and storage, drug preparation, transport, administration, waste management, cleaning procedures, accidental exposure and spill control; stage b: training, medical surveillance, documentation, biological monitoring.
Erce *et al*., 2016 [13]	Belgium	Parlamento Europeo	Governmental	English	Antineoplastic drugs and other hazardous drugs	Healthcare centres	General
Tomkins, 2015 [32]	USA	ONS, ASCO, HOPA	Professional	English	Hazardous drugs	Healthcare centres and home setting	General
Easty *et al*., 2015 [33]	Canada	CCO	Governmental	English	Antineoplastic drugs	Healthcare centres and home setting	Stage a: receiving and storage, drug preparation, transport, administration, extravasation (as a complication of administration), waste management, cleaning procedures, accidental exposure and spill control, patient care; stage b: training, medical surveillance, environmental monitoring, drug selection.
Spark *et al*., 2015 [34]	United Kingdom	Cardiff and Vale University Health Board, Gales	Governmental	English	Antineoplastic drugs	Healthcare centres	Stage a: receiving and storage, drug preparation, transport, administration, extravasation (as a complication of administration), waste management, accidental exposure and spill control, patient care; stage b: training; stage c: prescription, validation.
Guardino, 2015 [35]	Spain	INSHT	Governmental	Spanish	Antineoplastic drugs	Healthcare centres	Stage a: drug preparation; stage b: training.
Poveda *et al*., 2015 [36]	Spain	Grupo español de consenso	Professional	Spanish	Hazardous drugs	Healthcare centres	General
Goldspiel *et al*., 2015 [37]	USA	ASHP	Professional	English	Antineoplastic drugs and biotherapy agents	Regulatory agencies, manufacturers, healthcare centres and home setting	Stage a: receiving and storage, drug preparation, transport, administration, extravasation (as a complication of administration), accidental exposure and spill control, patient care; stage b: training, documentation, drug selection; stage c: prescription, validation, patient monitoring, manufacturing.
USP Convention, 2014 [79]	USA	USP	Governmental	English	Non-sterile drug preparations, including hazardous drugs	Healthcare centres	Stage a: receiving and storage, drug preparation, waste management; stage b: documentation, training; stage c: validation.
Health and Safety Executive, 2014 [38]	United Kingdom	HSE	Governmental	English	Antineoplastic drugs	Healthcare centres, home setting and veterinary clinics	Stage a: receiving and storage, drug preparation, transport, administration, waste management, cleaning procedures, accidental exposure and spill control, patient care; stage b: training, medical surveillance, documentation, biological and environmental monitoring.
British Columbia Cancer Agency, 2014 [39]	Canada	BCCA	Governmental	English	Antineoplastic drugs	Healthcare centres	Stage a: receiving and storage, drug preparation, transport, administration, waste management, accidental exposure and spill control, patient care; stage b: training, medical surveillance, documentation.
Arce *et al*., 2014 [40]	Spain	AMMTAS	Professional	Spanish	Antineoplastic drugs	Healthcare centres	Stage a: receiving and storage, drug preparation, transport, administration, extravasation (as a complication of administration), waste management, cleaning procedures, accidental exposure and spill control, patient care; stage b: training, medical surveillance, documentation; stage c: validation.
Casaus *et al*., 2014 [41]	Spain	MSSI	Governmental	Spanish	Drugs coHDounded at the Hospital Pharmacy Services	Healthcare centres	Stage a: receiving and storage, drug preparation, transport, waste management, cleaning procedures; stage b: training, documentation.
INSHT, 2014 [42]	Spain	INSHT	Governmental	Spanish	Biologic agents	Any workplace in which biological agents are handled, including healthcare centres	General
ASHP, 2014 [43]	USA	ASHP	Professional	English	Sterile drug preparations, including hazardous drugs	Healthcare centres	Stage a: receiving and storage, drug preparation, transport, waste management, cleaning procedures, accidental exposure and spill control; stage b: training, medical surveillance, documentation.
Alexander *et al*., 2014 [44]	Australia	WCMICS	Governmental	English	Monoclonal antibodies	Healthcare centres	Stage a: drug preparation, transport, administration, waste management, accidental exposure and spill control; stage b: training.
Siderov, Jim, 2013 [45]	Australia	COSA/CPG	Professional	English	Monoclonal antibodies	Healthcare centres	Stage a: drug preparation, administration, waste management, cleaning procedures.
PAHO, 2013 [16]	USA	PAHO-WHO	Non-governmental	English	Antineoplastic drugs	Healthcare centres	Stage a: receiving and storage, drug preparation, transport, administration, waste management, cleaning procedures, accidental exposure and spill control, patient care; stage b: training, medical surveillance, documentation.
INSHT, 2013 [46]	Spain	INSHT	Governmental	Spanish	Chemical agents	Any workplace in which chemicals are handled, including healthcare centres	General
ESOP, 2013 [47]	Germany	ESOP	Professional	English	Antineoplastic drugs	Manufacturers, healthcare centres and home setting	Stage a: receiving and storage, drug preparation, transport, administration, extravasation (as a complication of administration), waste management, cleaning procedures, accidental exposure and spill control, patient care; stage b: training, medical surveillance, documentation, biological monitoring; stage c: manufacturing, prescription, validation, patient monitoring.
The Quality Unit, NHS Scotland, 2012 [48]	United Kingdom	The Scottish Government	Governmental	English	SACT**	Healthcare centres and home setting	Stage a: receiving and storage, drug preparation, transport, administration, extravasation, waste management, cleaning procedures, accidental exposure and spill control, patient care; stage b: training, documentation; stage c: prescription, validation, patient monitoring.
INSHT, 2012 [49]	Spain	INSHT	Governmental	Spanish	Hazardous agents	Any workplace in which individual protection is necessary	General
Cohen, 2012 [50]	Spain	INSHT	Governmental	Spanish	Chemical agents	Any workplace in which chemicals are handled	General
Braun *et al*., 2012 [14]	USA	The Joint Commission	Non-governmental	English	Hazardous substances	Healthcare centres	General
Pérez *et al*., 2012 [51]	Switzerland	ESMO/EONS	Professional	English	Antineoplastic drugs	Healthcare centres	Stage a: extravasation (as a complication of administration); stage b: documentation; stage c: patient monitoring.
ASWCS, 2012 [52]	United Kingdom	ASWCS	Governmental	English	Antineoplastic drugs	Healthcare centres	Stage a: extravasation (as a complication of administration); stage b: training, documentation.
Goodin *et al*., 2011 [53]	International	Panel International de farmacéuticos	Professional	English	Oral antineoplastic drugs	Manufacturers, distributors, healthcare centres and home setting	Stage a: receiving and storage, drug preparation, transport, administration, waste management, cleaning procedures, accidental exposure and spill control, patient care; stage b: training; stage c: manufacturing, prescription.
Huber, 2010 [54]	USA	The Pennsylvania Patient Safety Authority	Governmental	English	Hazardous drugs	Healthcare centres and home setting	Stage a: receiving and storage, transport, administration, waste management, cleaning procedures, accidental exposure and spill control, patient care.
Cercós *et al*., 2010 [55]	Spain	GEDEFO	Professional	Spanish	Antineoplastic drugs	Healthcare centres	Stage a: accidental exposure and spill control; stage b: documentation.
Chaffee *et al*., 2010 [56]	USA	ASHP/UHC Pharmacy Council	Professional	English	Antineoplastic drugs	Healthcare centres	Stage a: receiving and storage, drug preparation, transport, administration, waste management, cleaning procedures, accidental exposure and spill control, patient care; stage b: training, medical surveillance, documentation.
ASWCS Network Nurse Group, 2010 [57]	United Kingdom	ASWCS	Governmental	English	Antineoplastic drugs	Healthcare centres	Stage a: receiving and storage, drug preparation, transport, administration, extravasation (as a complication of administration), waste management, accidental exposure and spill control, patient care; stage b: training, documentation; stage c: prescription, validation.
Carrington *et al*., 2010 [58]	Australia	COSA	Professional	English	Antineoplastic drugs and targeted therapy	Healthcare centres and home setting	Stage a: drug preparation, transport, administration, extravasation (as a complication of administration), patient care; stage b: training, documentation; stage c: prescription, validation, patient monitoring.
Russi *et al*., 2009 [59]	USA	ACOEM	Professional	English	Hazardous drugs	Healthcare centres	General
Jacobson *et al*., 2009 [60]	USA	ASCO/ONS	Professional	English	Antineoplastic drugs	Home setting	Stage a: drug preparation, administration, extravasation (as a complication of administration); stage b: training, documentation; stage c: prescription, validation, patient monitoring.
CAPhO, 2009 [61]	Canada	CAPhO	Professional	English	Antineoplastic drugs	Healthcare centres	Stage a: receiving and storage, drug preparation, transport, administration, waste management, cleaning procedures, accidental exposure and spill control; stage b: training, documentation; stage c: validation, patient monitoring.
INSHT, 2009 [62]	Spain	INSHT	Governmental	Spanish	Carcinogen or mutagen agents	Any workplace in which carcinogens or mutagens are handled	General
Shulman *et al*., 2008 [63,64]	USA	ASCO	Professional	English	Antineoplastic drugs	Healthcare centres and home setting	Stage a: drug preparation, administration; stage b: documentation; stage c: prescription.
Gallant *et al*., 2008 [65]	Canada	ASSTSAS	Professional	English	Hazardous drugs	Healthcare centres and home setting	Stage a: receiving and storage, drug preparation, transport, administration, extravasation (as a complication of administration), waste management, cleaning procedures, accidental exposure and spill control, patient care; stage b: training, medical surveillance, environmental and biological monitoring.
Connor *et al*., 2008 [66]	USA	NIOSH	Governmental	English	Hazardous drugs	Healthcare centres	General
ESOP, 2008 [67]	Germany	ESOP	Professional	English	Highly potent drugs	Healthcare centres and manufacturers	Stage a: transport.
Wengström *et al*., 2008 [68]	Switzerland	EONS	Professional	English	Antineoplastic drugs	Healthcare centres and home setting	Stage a: extravasation (as a complication of administration); stage b: documentation
USP, 2008 [69]	USA	USP	Governmental	English	Sterile drug preparations	Healthcare centres	Stage a: receiving and storage, drug preparation, transport, cleaning procedures; stage b: training, documentation, environmental monitoring; stage c: validation, patient monitoring, and sterilization.
Ohio Nurses Association, 2008 [70]	USA	ONA	Professional	English	Antineoplastic drugs and biologic agents	Healthcare centres and home setting	Stage a: administration; stage b: documentation.
SHPA Committee of Specialty Practice in Cancer Services, 2007[71]	Australia	SHPA	Professional	English	Antineoplastic drugs	Healthcare centres	Stage a: transport, waste management; stage b: training, documentation.
SHPA Committee of Specialty Practice in Cancer Services, 2007 [72]	Australia	SHPA	Professional	English	Oral antineoplastic drugs	Healthcare centres	Stage a: receiving and storage, drug preparation, transport, administration, waste management; stage b: training, documentation; stage c: validation.
Vulto *et al*., 2007 [73]	Europe	EAHP	Professional	English	Gene medicine	Healthcare centres	Stage a: receiving and storage, drug preparation, transport, administration, waste management, cleaning procedures, accidental exposure and spill control, patient care; stage b: training, medical surveillance, documentation.
Otero, 2007 [18]	Spain	MSC- USAL	Governmental	Spanish	High-risk medications	Healthcare centres	General
Connor *et al*., 2007 [74]	International	ISOPP	Professional	English	Hazardous drugs	Manufacturers, healthcare centres and home setting	Stage a: receiving and storage, drug preparation, transport, administration, extravasation, waste management, cleaning procedures, accidental exposure and spill control, patient care; stage b: training, medical surveillance, documentation, environmental monitoring, drug selection; stage c: validation, manufacturing.
Guardino *et al*., 2006 [75]	Spain	INSHT	Governmental	Spanish	Antineoplastic drugs	Healthcare centres	Stage a: receiving and storage, drug preparation, transport, administration, waste management, accidental exposure and spill control, patient care; stage b: training, medical surveillance.
ASHP, 2006 [76]	USA	ASHP	Professional	English	Hazardous drugs	Healthcare centres	Stage a: receiving and storage, drug preparation, transport, waste management, cleaning procedures, accidental exposure and spill control; stage b: training, medical surveillance.
Lymm *et al*., 2005 [77]	United Kingdom	NHS Grampian	Governmental	English	Antineoplastic drugs	Healthcare centres	Stage a: receiving and storage, drug preparation, transport, administration, extravasation, waste management, accidental exposure and spill control, patient care; stage b: training; stage c: prescription, validation.
SHPA Committee of Specialty Practice in Cancer Services, 2005 [78]	Australia	SHPA	Professional	English	Hazardous drugs	Healthcare centres	Stage a: receiving and storage, drug preparation, transport, waste management, cleaning procedures, accidental exposure and spill control; stage b: training, medical surveillance, documentation.
Burrougs *et al*., 2004 [11]	USA	NIOSH	Governmental	English	Hazardous drugs	Healthcare centres	Stage a: receiving and storage, drug preparation, transport, administration, waste management, cleaning procedures, accidental exposure and spill control, patient care; stage b: training, medical surveillance.

* Institutions: ACOEM American College of Occupational and Environmental Medicine; AMMTAS Asociación Madrileña de Medicina del Trabajo en el Ámbito Sanitario; ASCO: American Society of Clinical Oncology; ASCO/ONS American Society of Clinical Oncology/Oncology Nursing Society; ASHP American Society of Health-System Pharmacists; ASSTSAS Association paritaire pour la santé et la securité du travail du secteur affaires socials; ASWCS Avon, Somerset and Wiltshire Cancer Services, United Kingdom; BCCA British Columbia Cancer Agency; CAPhO Canadian Association of Pharmacy in Oncology; CCO: Cancer Care Ontario; COSA Clinical Oncology Society of Australia; COSA/CPG Clinical Oncology Society of Australia/Cancer Pharmacists Group; EAHP European Association of Hospital Pharmacists; ESMO European Society for Medical Oncology; EONS: European Oncology Nursing Society; ESOP European Society of Oncology Pharmacy; GEDEFO Grupo Español para el Desarrollo de la Farmacia Onocológica; HOPA Hematology/Oncology Pharmacy Association; HSE Health and Safety Executive, United Kingdom; INSHT Isnituto Nacional de Seguridad e Higiene en el Trabajo; ISOPP International Society of Oncology Pharmacy Practitioners; MSSI Ministerio de Sanidad, Servicios Sociales e Igualdad; MSC-USAL Ministerio de Sanidad y Consumo-Universidad de Salamanca; NHS National Health System; NIOSH National Institute for Occupational Safety and Health; ONA Ohio Nurses Association; ONS Oncology Nursing Society; OSHA Occupational and Safety and Health Administration; PAHO Pan American Health Organization; SEFH Sociedad Española de Farmacia Hospitalaria; SHPA Society of Hospital Pharmacists of Australia; UHC University Health system Consortium; USP The U.S. Pharmacopeia Convention; WCMICS Western and Central Melbourne Integrated Cancer Service.

**SACT: Systemic anticancer therapy.

## Results

Using the search criteria described, 1100 references were retrieved: 735 in MEDLINE, 183 in the Cochrane Library, 137 in Scopus, 3 in CINHAL, 42 in the Web of Science, and 49 provided by experts. No references were obtained from the search performed in the LILACS bibliographic database.

After applying the inclusion and exclusion criteria, reviewing the bibliographic lists, and consulting with experts (Fig 1), 61 documents were selected [11,13–16,18,24–79] (check Table 1).

The Kappa coefficient of the agreement in the selection of articles amongst the evaluators was 98.0% (p <0.001).

The 61 selected articles received an obsolescence rate, according to the Burton-Kebler Index, equal to 5 years, with a Price index of 45.9%.

The documents had been developed in 13 different countries: the USA (20 guidelines, 32.8%) and Spain (17, 27.9%) were largest producers of guides, followed by the United Kingdom (6, 9.8%), Australia (6, 9.8%), Canada (4, 6.6%), Switzerland (2, 3.3%), Germany (2, 3.3%) and Belgium (1, 1.6%). Likewise, other countries such as Sweden, Austria, Malaysia, France and Italy participated jointly in the elaboration of several documents both in the European scope (1, 1.6%) and at the international level (2, 3.3%).

**Fig 2:**
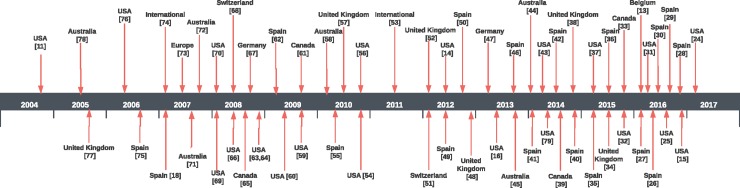
Timeline of guidelines developed.

Fig 2 presents the chronological development in a timeline figure to illustrate the sequence and development of guidelines over years and countries.

Regarding the language, 44 of the 61 (72.1%) references retrieved were written in English and 17 were (27.9%) in Spanish.

In relation to institutional affiliation, 36 different organizations were identified: 15 agencies or governmental institutions authored 30 (49.2%) documents, 19 professional societies developed 29 documents (47.5%), and 2 non-governmental agencies published 2 (3.3%) guides; see Table 1.

### Scope

Most of the documents (38, 62.3%) focused their recommendations on the handling of hazardous substances in health centres, and 12 (19.5%) took into account the home setting. In 5 (8.2%) documents [42,46,49,50,62], the scope was general (i.e., the application area of the guidelines is not specific but applies to any sector in which such hazardous substances are handled), and 6 (9.8%) guides included, in addition to the health area, recommendations for other sectors, such as the pharmaceutical industry, regulatory agencies and veterinary clinics [37,38,47,53,67,74].

### Type of substance

Twenty-four documents approached HD-related recommendations (39.3%) [11,14,15,25–32,36,43,54,56,59,65–67,69,74,76,78,79], whereas 27 studies (44.3%) focused on antineoplastics (specific for anticancer drugs) [13,16,24,33–35,37–40,47,48,51–53,55,57,58,60,61,63,64,68,70–72,75,77]. In addition, 10 guidelines (16.4%) addressed other types of substances, such as monoclonal antibodies, gene medicine and other chemical and biological agents [18,41,42,44–46,49,50,62,73].

### Stages in the management process for dangerous drugs

Amongst all the retrieved guidelines, 14 (23.3%) references considered all the stages of the manipulation process in which there is a risk of exposure (stage a); the extravasation stage was considered a part of the administration stage since it is a complication of this [11,15,16,26,33,38,40,47,48,53,56,65,73,74]. Of these 14 guides, 13 also considered stages in the handling process without risk of exposure: drug selection and commercial presentations [26,33,74], staff training and/or patient education [11,15,16,26,33,38,40,47,48,53,56,65,73,74], documentation [15,16,26,38,40,47,48,56,73,74], medical surveillance [11,15,16,26,33,38,40,47,56,65,73,74] and environmental and/or biological monitoring [15,33,38,47,65,74]. Furthermore, 6 of the 14 references also addressed complementary stages: prescription [47,48,53], validation [40,47,48,61,74], patient monitoring [47,48,61], and manufacturing [47,53,74].

Only one guide, the ISOPP Standards of Practice [74], addressed all stages of the process of handling hazardous substances (stages with and without risk of exposure).

No standardized systems to ensure quality management, traceability of processes, and minimization of risks associated with these drugs were found. There was no mention in the reviewed guidelines of any computerized system to ensure the proper management of the entire HD process.

Twelve (20%) of the selected documents did not include specific stages of the manipulation process. Their content was of a general nature, describing transversal actions that can affect all stages of the process, general prevention measures, and strategies, guidelines or policies to be followed [13,14,18,32,36,42,46,49,50,59,62,66].

The most described stages were elaboration (41 guides, 67.2%), staff training and/or patient education (38 guides, 62.3%), and administration (37 guides, 60.7%). The stages that were less frequently addressed were cleaning and decontamination procedures (26 guidelines, 42.63%), patient care (24 documents, 39.3%), and medical surveillance (18 documents, 29.5%) [Table 2].

**Table 2:** Rate of each stage in the selected documents and its classification regarding exposition.

Stage	n°	%	References	Stage type
Receiving and storage	33	54.1	[11,15,16,24–26,30,31,33,34,37–41,43,47,48,53,54,56,57,61,65,69,72–79]	a
Drug preparation	41	67.2	[11,15,16,24–31,33–35,37–41,43–45,47,48,53,56–58,60,61,63–65,69,72–79]	a
Transport	36	59.0	[11,15,16,24,26,28,29,31,33,34,37–41,43,44,47,48,53,54,56–58,61,65,67,69,71–78]	a
Administration	37	60.7	[11,15,16,24–27,29,31,33,34,37–40,44,45,47,48,51–54,56–58,60,61,63–65,68,70,72–75,77]	a
Extravasation	16	26.2	[24,33,34,37,40,47,48,51,52,57,58,60,65,68,74,77]	a
Patient care	24	39.3	[11,15,16,24–26,33,34,37–40,47,48,53,54,56–58,65,73–75,77]	a
Waste management	34	55.7	[11,15,16,25,26,28,29,31,33,34,38–41,43–45,47,48,53,54,56,57,61,65,71–79]	a
Exposure and spill control	31	50.8	[11,15,16,25,26,28,29,31,33,34,37–40,43,44,47,48,53–57,61,65,73–78]	a
Cleaning procedures	26	42.6	[11,15,16,25,26,28,29,31,33,38,40,41,43,45,47,48,53,54,56,61,65,69,73,74,76,78]	a
Drug selection	4	6.6	[26,33,37,74]	b
Personnel training and/or patient education	38	62.3	[11,15,16,24,26,28,29,31,33–35,37–41,43,44,47,48,52,53,56–58,60,61,65,69,71–79]	b
Documentation	30	49.2	[15,16,24,26,31,37–41,43,47,48,51,52,55,56,58,60,61,63,64,68–74,78,79]	b
Medical surveillance	18	29.5	[11,15,16,26,31,33,38–40,43,47,56,65,73–76,78]	b
Environmental and/or biological monitoring	8	13.1	[15,31,33,38,47,65,69,74]	b
Prescription	11	18.0	[24,34,37,47,48,53,57,58,60,63,64,77]	c
Validation	14	23.0	[34,37,40,47,48,57,58,60,61,69,72,74,77,79]	c
Patient monitoring	8	13.1	[37,47,48,51,58,60,61,69]	c
Manufacturing	4	6.6	[37,47,53,74]	c
Sterilization	1	1.6	[69]	c

(a) Stages with risk of exposure

(b) Stages without risk of exposure

(c) Complementary stages

## Discussion

The high number of retrieved guidelines shows the existing concern regarding exposure to HDs and the safe management of these substances. However, unexpectedly, there was only one international consensus document that tackles the entire process of HD manipulation, and there were no computerized systems recommended to guarantee proper management of the HD process.

This study shows that obsolescence is very present. Half of the recovered guidelines were published in the last 5 years, a larger ratio than other previously published papers in the health sciences and hospital pharmacy environments [80,81]. These results support the high interest that the study of HD management is experiencing in recent years.

It is not surprising that the United States is the place of origin of most guidelines since it is the country with the highest scientific production. It must be borne in mind that eight of the top ten universities in the world are in the USA, which continues to be the world leader in science and innovation [82]. However, European countries as a whole are the promotors of the largest scientific production in this context, a phenomenon previously observed by Hon et al. [83].

Although works from both continents have the same validity, it is important to note that the recommendations contained in documents from the US may not be transferable to Europe, mainly due to differences in the regulatory framework of the different countries. Therefore, given the relevance of the issue to workers' health, more initiatives at an international level should be performed to harmonize standards and unify the legal framework as far as possible.

The English language was chosen for publication in most articles since a different language could have a negative impact on visibility and citability. In addition, the number of English-language journals contained in the considered databases is currently very high [82,84].

The institutional affiliation reflects the commitment of the agencies involved in the HD process. Despite multiple efforts made worldwide to establish standards in the management of MP, the safety of HD manipulation is an unresolved issue, which concerns governments and professional societies worldwide because there is a wide range of variants amongst the different guidelines. This conclusion has already been highlighted by other authors [85]. Proof of this is shown in the recent publication from the European Parliament [13], which reflects the preoccupation of addressing this issue.

It is important to emphasize that professional societies were linked entirely to the health field. Involved governmental agencies depend as much on health administration as on labour administration.

### Scope of application

More than one-half of the retrieved documents were targeted exclusively at health centres, and only one-fifth addressed the dangers that might occur at home. With the growing number of oral medications being approved in cancer treatment, the potential for the long-term administration of these drugs to cancer patients is expanding. The use of these drugs at home has the potential to expose family members and caregivers to them, either through direct contact with the drugs or indirectly by exposure to the parent compounds and/or their active metabolites in contaminated patients’ waste [86]. This is relevant since the manipulation of HD in this context requires an adequate strategic plan of intervention, monitoring and tracking.

It is important to keep in mind that exposure to risks for professionals, patients and their relatives may not only occur during the stay in the hospital; these risks also occur when HDs are used at home, where the precaution of the patients and caregivers can be more relaxed [20]. Although hospital infrastructure is no longer necessary in the Hospital at Home (Hospital Home Care Services), patient care remains complex [87].

### Type of substance

The different guidelines use a heterogeneous terminology when referring to the types of substances addressed, mainly due to the evolution of the definition of HD. Therefore, it is not surprising that most of the guidelines limit their recommendations to antineoplastic or cytotoxic drugs (both terms commonly used interchangeably in the literature to refer to drugs used in the treatment of cancer [85]). The main reason for this is linked to the fact that the risks associated with its manipulation are clearly defined because they are usually prepared in centralized pharmacy aseptic units.

However, according to the NIOSH definition [11] in 2004 that includes antineoplastic and other non-antineoplastic drugs, and particularly after the later update in 2014 of the NIOSH document [12], in which HDs are classified into three lists, a growing trend in the publication of guidelines tackling the concept of HDs can be noted. These new guidelines use a broader and more inclusive concept of HDs.

Conversely, guidelines addressing drugs widely used in the oncology field were taken into account (monoclonal anti-blockers [44,45], genes medicines [73]), which were not initially considered by NIOSH as HDs and whose manipulation has generated uncertainty and variability in clinical practice.

Likewise, guidelines covering agents that comprise HDs were contemplated since they deal more generally with the handling of dangerous substances (chemical agents [46,50], carcinogenic and/or mutagenic [62], biological agents [42], drugs prepared in pharmacy services [41] and high-risk medications [18]).

### Stages in the management process of hazardous drugs

The guidelines were very heterogeneous regarding the stages described, likely because there is no international consensus on the phases that comprise the HD manipulation process. To illustrate this feature, we can note that although European authors consider actions such as staff training and/or patient education, medical surveillance and environmental and/or biological monitoring as stages of the process [26,65,74], their American colleagues consider these actions as administrative controls [88].

In general terms, the most frequently mentioned stages were those classified by the authors as stages with a risk of exposure. This is reasonable because elaboration and administration, along with waste management, are considered by NIOSH as the riskiest phases of the process for staff [11].

Special mention should be made about the stages regarding staff training and/or patient education and documentation, both of which are transversal stages affecting all phases of the process. Even without the risk of exposure, these stages were considered more profusely than other stages without risk of exposure, such as the stages of procedures for cleaning and care of the patient. This shows that both stages are fundamental to guarantee the quality of the HD handling process, in which all steps must be performed by qualified staff [89], following standard protocols and recording all the operations performed throughout the entire life of the HD. Through this recording, the full traceability of the process and the supervision of all the involved stages is facilitated. In this way, it will be possible to evaluate the system as a whole and to establish to which extent the actions comply with the established standards to indicate the points with a margin for improvement and to prevent hazards.

The verification procedures are necessary to evaluate the efficiency of a process and to ensure that there is an adequate control of all the possibilities of risk [90].

A benefit derived from computer-based systems is the support of repositories for the generated records that allow linking, verifying and evaluating data at any time, guaranteeing excellence in management control and traceability [91, 92]

Just one guide [74] covering all the stages of the HD handling process could be identified. This may have occurred because most recovered guides do not include one or several stages in which there is no risk of exposure. Nevertheless, these stages are not of lesser relevance and should also be addressed.

There was a general lack of environmental and/or biological monitoring in the guidelines. This may be due to the limited existence of analytical methods for the quantification of most HDs, both in biological and environmental samples. Currently, there are standardized methods to measure the concentration of just some anticancer drugs, whereas many others are available only in a research setting [65]. Conversely, in most cases, there are no reference standards for environmental exposure, so the interpretation of the results must be performed with caution, and measures must be taken to reduce exposure "as low as reasonably achievable" (ALARA) [65, 93].

## Limitations

This work only took into consideration documents provided in English or in Spanish. This set of languages provides a joint coverage of more than 90% of the existing literature in this area [80–82] and includes coverage of many countries (organizations from many countries publish their documents in both English and their official language). Nevertheless, to ensure the best possible identification of main stages, guides in other languages were also consulted. Thus, guides in French [94,95], German [96] or Italian [97–99] were considered, but, as the reader may note in Table 1, no new stages were identified.

The high rate of non-relevant articles in relation to the final selection made can be considered a possible limitation of this review. This may be due to the lack of descriptors (MeSH) specific to the "hazardous drug" concept. The lack of a specific MeSH term forced us to conduct the MEDLINE search in text format using the title and abstract fields. Moreover, the Web of Science and Scopus databases do not have a thesaurus, so the query must be performed in text format using the title, abstract and keywords field, preventing the use of descriptors. This disturbing "noise" from the retrieval point of view has been previously observed in other systematic reviews [100,101]. Likewise, there was publication bias because most references are part of the grey literature since they are reports produced by institutions of different natures and therefore are not indexed in bibliographic databases with scientific content [102].

## Conclusions

Based on the above findings, we can conclude that no standardized informatics system was found to ensure quality management, traceability of processes and minimization of risks associated with these drugs. In the considered guidelines, no mention of computerized systems that guarantee the correct management of the HD process was identified.

From the authors’ point of view, it would be convenient to be at the disposal of ICT-based tools that allow a simple and complete configuration of management systems to tackle the prevention of risks associated with HDs. Moreover, further works and specific developments regarding the management and traceability of HDs that allow for their monitoring and evaluation must be generated.

## Supporting information

S1 Checklist PRISMA checklist. (PDF)

S1 File MEDLINE (via PubMed) search strategy. (DOCX)

S1 Certificate Certificate AMJ. (PDF)
