# Supplementary material for: Guidelines for safe handling of hazardous drugs: A systematic review
Source: PLoS One. 2018 May 11;13(5):e0197172. doi: 10.1371/journal.pone.0197172 (PMC5947890; doi:10.1371/journal.pone.0197172)
Supplement: S1 File — (DOCX) [file pone.0197172.s002.docx]

**S1 File. MEDLINE (via PubMed) search strategy**

Antineoplastic Agents"[Mesh] OR "Antineoplastic Agents"[Title/Abstract] OR “Antineoplastic Drugs”[Title/Abstract] OR “Antineoplastics”[Title/Abstract] OR “Chemotherapeutic Anticancer Drug”[Title/Abstract] OR “Antitumor Drugs”[Title/Abstract] OR “Cancer Chemotherapy Agents”[Title/Abstract] OR “Cancer Chemotherapy Drugs”[Title/Abstract] OR “Chemotherapeutic Anticancer Agents”[Title/Abstract] OR “Anticancer Agents”[Title/Abstract] OR “Antitumor Agents”[Title/Abstract] OR "Hazardous Substances"[Mesh] OR "Hazardous Substances"[Title/Abstract] OR “Hazardous Materials”[Title/Abstract] OR “Hazardous Chemicals”[Title/Abstract] OR “Environmental Toxic Substances”[Title/Abstract] OR “Toxic Environmental Substances”[Title/Abstract] OR “Biohazards”[Title/Abstract] OR "Cytostatic Agents"[MeSH Terms] OR "Cytostatic Agents"[Title/Abstract] OR “Cytostatics”[Title/Abstract] OR “Cytostatic Drugs”[Title/Abstract] OR “Hazardous Drugs”[Title/Abstract] OR “Chemotherapy” [Title/Abstract] OR “Chemotherapeutic Agents” [Title/Abstract] OR “Chemotherapeutic Drugs”[Title/Abstract] OR ”Cytotoxic Drugs”[Title/Abstract] OR “Cytotoxics”[Title/Abstract] OR “Antineoplastic medications”[Title/Abstract] OR “Anticancer Drugs”[Title/Abstract] OR “Highly potent drugs”[Title/Abstract].
